# 

**Published:** 1887-07

**Authors:** 


					﻿American Dental Association Meeting.
The annual meeting of this body will be held at Niagara Falls
on the first Tuesday of August prox. According to the decision ■
of last year it would have been held at Ashville, N. C. But for
reasons with which all are probably familiar, it was thought best
to change to another place, and Niagara was selected. Many
thought the time should also have been changed, but those having,
the matter in charge decided otherwise ; and now it is the duty
of all members' and friends of the Association to make the meet-
ing as large and successful as possible.
It is true that the meeting of the International Medical Con-
gress, which will be held a month later will have attractions for,,
and be interesting to many of the members of the American
Dental Association, but to all such for whom it is possible we
say most earnestly, attend both of these meetings ; such a con-
currence has never taken place before in this country, and may
never,   again.
And now let there be an extra effort put forth to sustain the
American Dental Association.
Rates to Niagara Falls.
The fare for those going to the American Dental Association,
will be from Cincinnati by the New York, Pennsylvania, &
'Ohio R. R., $14.75 the round trip. This is just about one full
fare ; certainly as cheap as any one could ask. At the various
points on the way the same reduction of rates will be given, viz :
from Dayton, the round trip will be $13.50 ; from Springfield,
$13.00; from Mansfield, $10.75. These tickets will be good
until October 31. No certificates are required. At Niagara the’
International Hotel will be the headquarters for the members ;
and the same quarters have been obtained for the accommoda-
tion of the meeting as last year ; which is about all that can be
desired in that direction. It is hoped that all will come with full
intent to make the meeting an interesting and profitable one.
•Give politics the go-by this time.
Cincinnati, O., June 27, 1887.
The National Association of Dental Faculties will meet at the
Ebbitt House in Washington, D. C., on Saturday, September
3, 1887, at 10 o’clock a. m. By Order of the Executive Com-
mittee.	C. N. Peirce, President.
H. A. S^th, Secretary.
The American Dental Association.
Chicago, June 25, 1887.
AU lailroads west of Chicago, and from Chicago leading 1o
Niagara Fails will larry membeas and isleaates of the American
Deatal Astooiction at one and ofe-thicri lares.
READ THE	lA ItEEUEEV.
“ Each dslegale neist purchase a hast oiert ticket lo the (Dlaca of
measiag tan tviiicii he iwll pay the irguler fare and upon request
the ticket agaat will latte lo lim a oasSiacale of aueh purchase.
If ahroughlicketi camof be profuerd i1 lhe lthriiag poiat
dstegales wUl purchase lo lhe ne.nt oaneeaicgt pofct wVerr lueh
tieough 1ickeri oan le odeamed aad l'e-puechare lhrough lo pOace
od meatiBg, rs-£uestiag a tat■Siarale from lhe 1icket lguat o1 lhe
point weeae repurchart lc
r^icketi laf lhe	lauraey wiH beooid by lhe Cicket lguat
ar lhe pOace oO mertiag o1 ont^-tiiirsi lhe tighett Clleilcd lare mly
to lhooe hoieicg cartiacariJs ligaed by lhe Cicket agct i1 lhe
point weear lheough 1icke- lii pOace of merSiag lver pc^rr'anrstai
aad 1anuni^-s^il^re-g.i by lhe Serre-hly on diab of lhe Contention,
cariinaing 1hal lhe bondsa ler bem la artendance upon lhe Con-
vention.”
Ticket are good, goicg, lhree days beaare lhe meaSing ond
^0™^, lhree lsys afths lci 1iaeeIel^^i<de
A. lW. Harlan, 10ia^l'ieltl Exec. Com.
P. S. EquaUy lavarable catet are	lrem liee Soulh
and East
The Southern Dental Association.
The annual meeting of this body will be held at Old Point
Comfort, Va., on Tuesday, August 30. There are several con-
siderations to make this a large and interesting meeting. In the
first place no more desirable place could be found in the country
than this ; because of its attractions, it is very popular.
A good degree of effort and energy is being put forth for secur-
ing a good meeting; the indications thus far are that this will
be the largest meeting of this association ever held.
Another favorable circumstance is the time, being the week
before the meeting of the Congress ; many will make it a point
to attend both gatherings. All visitors may rest assured of a
most hearty welcome, and the warmest hospitality.
We may suggest that all who can should attend this ' meeting
as a preparation for that of the next week.
Association of the Boards of Dental Examiners.
The annual meeting of this body will take place at Niagara
Falls on Monday, August 1, at 3 o’clock p. m. It is very desir-
able and important that all State Boards should be represented
at this meeting, as important interests will come up for consider-
ation.
The action had at the recent meeting of the American Medical
Association bearing upon the status of dental practitioners, will
of necessity, more or less influence our educational institutions
and this will bring additional responsibilities upon this Board.
The power of this body is very great, and it is of the utmost
importance that it be exercised with care and wisdom, and in
such a manner as shall promote the best interest of all concerned.
We will hope to see every Board in the country represented
at this meeting.
National Association of _ Dental Examiners.
The annual meeting of the National Association of Dental
Examiners will be held at Niagara Falls, on Monday, August 1,
1887, at 3 p. m.	Fred. A. Levy, Sec’y, Orange, N. J.
The DENTAL REGISTER,
A Monthly Magazine Devoted to the Interests of the
DENTAL PROFESSION.
"Terms Reduced to $2.00 Per Tear. Single Copies, 25 Cents.
EDITED BY PROF. J. TAFT, D.D.S.
---—AND—-------
Puhlishea by SAH'L &. CROCKED & CO., Ohio Dental and Surgical Depot
The volume commences in January and closes in December.
The Register being issued monthly is always fresh, therefore Indispensable
to the practitioner who desires to keep posted up with the new inventions and
improvements which are daily developed. Neither expense nor effort will be
spared to give value and variety to its contents. Articles will be illustrated with
well executed engravings whenever they will add to the interest or assist to ex-
planation.
Its extensive circulation and frequency of issue make it one of the BEST DEN-
TAL ADVERTISING MEDIUMS in the United States.
All subscriptions must begin with January or July.
Local or special notices, five lines or less, will be inserted for $3 each insertion ;
over five lines, 50 cts. per line.
All advertising bills payable in advance, or at furthest, after the issue of the
first number containing the advertisement. All transient advertisements to be
paid for in advance.
^CONTRIBUTIONS SOLICITED.
-'Exclusively Editorial Communications should be addressed to the Editor.
The latest number of the Register may be ordered with or without other goods
»t any time, and all letters should be addressed to
SAM’L A. CROCKER & CO., Ohio Dental and Surgical Depot, Cincinnati, 0.
				

